# Correlation between Corpus Callosum Sub-Segmental Area and Cognitive Processes in School-Age Children

**DOI:** 10.1371/journal.pone.0104549

**Published:** 2014-08-29

**Authors:** Martha Beatriz Moreno, Luis Concha, Leopoldo González-Santos, Juan Jose Ortiz, Fernando Alejandro Barrios

**Affiliations:** Universidad Nacional Autónoma de México, Instituto de Neurobiología, Querétaro, México; University of Tuebingen Medical School, Germany

## Abstract

We assessed the relationship between structural characteristics (area) and microstructure (apparent diffusion coefficient; ADC) of the corpus callosum (CC) in 57 healthy children aged 7.0 to 9.1 years, with diverse cognitive and academic abilities as well as executive functions evaluated with a neuropsychological battery for children. The CC was manually delineated and sub-segmented into six regions, and their ADC and area were measured. There were no significant differences between genders in the callosal region area or in ADC. The CC area and ADC, mainly of anterior regions, correlated with different cognitive abilities for each gender. Our results suggest that the relationship between cognitive abilities and CC characteristics is different between girls and boys and between the anterior and posterior regions of the CC. Furthermore, these findings strenghten the idea that regardless of the different interhemispheric connectivity schemes per gender, the results of cognitive tasks are very similar for girls and boys throughout childhood.

## Introduction

The main function of white matter (WM) is to communicate between cortical and subcortical areas, forming the basis of large-scale neuronal networks. The CC is the largest interhemispheric connection and is composed of millions of neural fibers [1]. Structural MRI studies have found a large range in size of the CC in healthy subjects [2], a variability that is mirrored in the proportion of fibers directed towards particular cortical regions in healthy adults [3]–[4]. Some researchers [5]–[9] have suggested that this anatomical variability in the CC may be associated with intellectual capacities, e.g., a larger CC cross-sectional area may result in more efficient interhemispheric communication and better intellectual performance. Indeed, a positive correlation has been found between the width of the CC, particularly its posterior and isthmus portions, and the intelligence coefficient (IQ) in healthy adults, which led to the conclusion that more efficient interhemispheric communication may result in better integration and processing of information [4]. In a recent post-mortem study, the width of Albert Einstein's CC was compared to that of young adults and senior citizens. A greater width was found in Einstein's CC in all regions, especially in the splenium, as compared to the youngest controls. Hence, Einstein's intellectual abilities may not only be associated with cortical folding and cytoarchitecture in certain regions of his brain, but also with the greater width of his CC, likely underlying an efficient interhemispheric communication [10]. In pediatric populations the hypothesis that a larger CC results in increased cognitive capacity has been controversial. MRI studies in children and adolescents have found negative correlations between the area of some CC regions and measures of intellectual ability [6]–[9]. Hutchinson et al. [6], Allin et al. [7], and Ganjavi et al. [8] found negative correlations in adolescents between the area of the CC and their IQ. Luders et al. [9] and Ganjavi et al. [8] concluded that the correlations between the CC and cognitive abilities depend on sex and age. Gender has been shown to differentially impact the growth pattern of the CC [5], while significant differences in the thickness of the CC have been described as a function of age, from 5 to 18 years [9]. Luders et al. [9], observed that not all CC regions grow uniformly in children during development nor between genders, and they showed that callosal thickness increased at specific rates for each gender and CC region, with the exception of the CC frontal region in girls. The relation between the morphology of the CC and intellectual abilities has been described as dynamic during brain maturation for male subjects, but not among females [9], suggesting either a gender difference in the neuronal pruning process resulting in improved interhemispheric communication or a decrease in myelinated axons (with greater conduction speed), corresponding to a better intrahemispheric capacity, thereby reducing the need for interhemispheric exchange.

In addition to structural CC studies, diffusion weighted imaging investigations have also focused on microstructural properties estimated through the apparent diffusion coefficient (ADC). Using water diffusion as a probe of microstructure is a valuable tool for the in vivo evaluation of white matter tissue [11]–[12]. In general, increased ADC values are typically observed in tissues with few obstacles to the free diffusion of water molecules; in white matter such tissues are axonal membranes and myelin sheaths. This technique has demonstrated that the structural integrity of the CC is associated with intellectual abilities in healthy subjects and those with certain neurological conditions, such as developmental disabilities, schizophrenia, autism, and Tourette's syndrome, among others, where the microstructure of the CC is compromised [13]–[17]. In healthy adolescents and young adults, Hutchinson et al. [6] found a negative correlation between IQ and ADC values in the genu.

Gender differences in the CC have also been investigated, and some findings related to the micro- and macro-structure of gender differences in adults have been questioned [18]–[26]. Neurodevelopmental studies have demonstrated gender growth pattern [24]–[26] and total volume differences in the brain and CC. The brains of girls reach their adult volume, on average, at 10.5 years of age, while those of boys do so at 14.5 years. In contrast, WM grows faster in boys than in girls, particularly in the teenage years [24]–[25]. Such temporal differences in development may explain why various correlations between the CC and cognitive abilities depend on sex and age [9], [27]. Studies linking intellectual abilities and CC properties have been made mainly using intelligence tests (IQ tests), and reports using other kinds of evaluations like neurodevelopmental tests are lacking.

Based on the importance of WM integrity in cognitive functioning and considering the gender differences in neurodevelopment of WM [9], [19], [22]–[23] and the differential associations between IQ and CC size reported in previous studies, in the present study we asked whether the cross sectional area of different CC regions and their microstructure (assessed with ADC) correlated with cognitive abilities and whether gender differences affect these associations. In order to identify such associations, we performed an extensive neuropsychological evaluation, the Neuropsychological Assessment of Children (ENI, from the Spanish “Evaluación Neuropsicológica Infantil”) [28] (Table S1). Our hypothesis was that the regional area and ADC of the mid-sagittal plane of the CC in healthy children between 7 and 9 years old correlates with their performance in cognitive, executive, and academic performance abilities, with differences between genders.

## Methods

### Subjects

A general invitation explaining the characteristics and inclusion criteria was made through local grade schools, and parents were asked to talk with their children about whether they wanted to participate in interviews and MRI sessions. For those cases when both parents and the child wanted to participate, a session was arranged to give them more detailed information and a careful explanation of the experimental methodologies; questions from parents and children were encouraged, and informed consent forms were signed by both parents. Structured interviews, a general medical examination, neuropsychological exploration (ENI) [28]–[30], and MRI studies were performed on 120 healthy children evenly distributed between 7 years and one month and 8 years and 11 months old. The inclusion criteria were: pregnancy to term, no neurological impairment, no neurodevelopmental, learning, and/or language problems, not having repeated any school year, and being healthy. Not all children finished the ENI test, and many subjects were dropped because their MRI presented motion artifacts. Thus, from the original 120 subjects studied, the results from 57 children (25 boys and 32 girls) were suitable for further analyses (Table 1). Of these 57 MRI, 14 were acquired using a 1.0 T MR scanner (6 girls and 8 boys) and 43 with a 3.0 T scanner (25 girls and 18 boys). It is common to have a large proportion of dropouts in pediatric studies due to motion resulting from anxiety and general nervousness. For studies with 5 year olds, the exclusion rate has been estimated to be approximately 50%, which diminishes with age [31]; our rate of 52.5% likely resulted from motion during one of the MRI acquisition paradigms and from the length of the ENI test, which may require a couple of sessions to finish.

**Table 1:** List of subjects by age and sex.

AGE	BOYS	GIRLS	TOTAL	MEAN
YEARS.MONTHS				
7.0–7.3	4	3	7	7.1
7.4–7.7	1	1	2	7.5
7.8–7.11	3	6	9	7.69
8.0–8.3	9	2	11	8.16
8.4–8.7	2	7	9	8.5
8.8–8.11	2	6	8	8.6
9.0–9.3	2	3	5	9.1
9.4–9.7	0	4	4	9.6
9.8–9.11	1	1	2	9.10
AVERAGE				8.3
TOTAL	24 (42.1%)	33 (57.8%)	57	SD = 0.74

*Ethics Statement:* Both parents of each child answered structured and clinical interviews before any study was done to their child, and they read and signed a letter of informed consent according to the methods and letters authorized in the project entitled Study of Cerebral Magnetic Resonance Imaging for the Creation of a Children's Atlas [Estudio de resonancia magnética del cerebro para la creación de un atlas infantil], which follows the principles expressed in the Declaration of Helsinki and was authorized by the Bioethics Committee of the Neurobiology Institute [Comité de Bioética del Instituto de Neurobiología].

### Neuropsychological battery

We used an integrated battery of several tests as an initial exclusion evaluation. We applied Bender's Visual-Motor Gestalt Test, which estimates visual motor development and reflects the mental development of the child [29], and the MINI Kid for children and adolescents [30] that evaluates the main psychiatric disorders of infancy based on the DSM-IV and CIE-10 classification. This initial evaluation allowed us to detect neurological or psychiatric risks or alterations. Only those participants with typical development were tested with the ENI, which evaluates diverse cognitive abilities grouped into three domains: executive functions, cognitive functions, and academic performance (Table 2). The ENI requires 300 minutes on average per subject, and its main difference from other more commonly used tests, such as Wechsler's Intelligence Scale for Children (WISC) [32]–[33], is that the ENI is a neuropsychological evaluation, not an IQ test. Nonetheless, there is overlap between the tests, and the two are highly correlated [28]. The cognitive function domain of the ENI explores basic cognitive processes like attention, perception, language, and metalanguage as well as spatial, conceptual, and constructional abilities. The executive function domain explores cognitive fluidity (verbal and graphical) and cognitive flexibility (organization, categorization, and perseverance), and the academic domain explores the child's reading, writing, and math execution. Each of the 33 subtests that make up the ENI is evaluated with a different task, making the ENI a very complete instrument for neuropsychological exploration. The description of each ENI subtest can be found in Table S1. The results section will include descriptions of all subtests from which significant results were obtained.

**Table 2:** Test and subtest for each domain of neuropsychological assessment of children (ENI).

COGNITIVE FUNCTIONS		ACADEMIC PERFORMANCE		EXECUTIVE FUNCTIONS	
TEST	SUBTEST	TEST	SUBTEST	TEST	SUBTEST
Construction Abilities	With objects		Precision		Verbal
		Reading	Comprehension	Cognitive fluency	Graphic
			Speed		
	Graphic		Precision		Perseverance
Memory	Verbal and Visual	Writing	Narrative composition	Cognitive Flexibility	Categorical organization
	Codified and differentiated		Speed		
	Tactile		Counting		Designs
	Visual		Numerical management		
Perception		Planning and organizing	Calculus	Planning and organizing	
	Auditory		Logical-mathematic		Movements
			Reasoning		
Metalanguage					
Spatial abilities					
Attention	Visual				
	Auditory				
Conceptual abilities					

### Magnetic Resonance Imaging

Images were acquired using two MR scanners, a 3.0T G.E. Discovery MR750 (General Electric, Waukesha, WI) with a 16-channel-array head coil, and a 1.0T Philips Intera (Philips, Best, Netherlands) with a quadrature coil. The 1.0T scanner was decommissioned while this project was ongoing. Of the children included in the analyses, 14 were scanned on the 1.0T scanner, while the remaining 43 where imaged with the 3.0T scanner. Images acquired at 1.0T included a high-resolution structural 3D T1-weighted fast-field echo pulse sequence with 2×2×2 mm^3^ spatial resolution (TR/TE = 25/6.9 ms); flip angle = 30.0°, 143 slices, acceleration = 1.0, without inversion time. DWI images of the 1.0T scanner were obtained in three orthogonal planes with b = 1000 s/mm^2^, along a reference volume (b = 0 s/mm^2^) with 3×3×3 mm^3^ spatial resolution (TR/TE = 7324.5/104 ms), 39 slices, acceleration = 4.0. Images acquired in the 3T scanner included a high-resolution structural 3D T1-weighted SPGR pulse sequence with 1×1×1 mm^3^ spatial resolution (TR/TE = 8.1/3.2 ms); flip angle = 12.0°, matrix 256×256. DWI images were acquired on the 3T scanner using an EPI sequence (TR/TE = 7000/81 ms), with b = 1000 s/mm^2^ and 35 diffusion gradient directions, with 2×2×2 mm^3^ spatial resolution, 58 slices, matrix 128×128. While data from the 3.0T scanner was suitable for diffusion tensor modeling, in order to combine both data sets, only the average ADC was evaluated. All the images were stripped of any personal information and transferred offline for analysis.

### Image and statistical analysis

**Figure 1:**
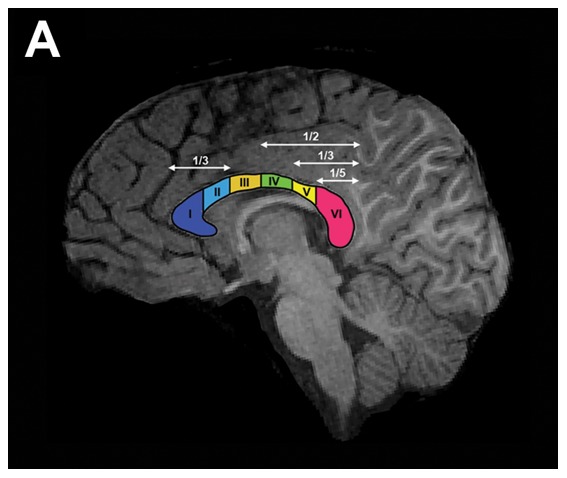
Corpus callosum segmentation as proposed by Park et al., (2008). A) CC delineated on T1w of sample subject.

Briefly, all images were registered to the MNI152 atlas using an affine transformation with 6 degrees of freedom using FSL's tools [34]–[35]. The re-aligned images were grouped and then for each subject, the CC was delineated by hand in the medial sagittal slice of the high resolution T1w and the DW images, separately. Next, the outline of the CC of each subject was sub-segmented automatically into six regions, according to the scheme proposed by Park et al. [4] (Figure 1). Total CC area at the mid-sagittal plane was measured and the area for each of the six CC regions were corrected for brain-size by dividing them with the total mid-sagittal plane area, scaling to the total cross section area of the brain (TAB). ADC values for each of the six CC regions were covaried with the total area of the CC. Area and ADC regional values were correlated with group and individual ENI evaluations using the general linear model (GLM), and corrected for multiple comparisons with a permutation test running the R package lmPerm (lmPerm v1.1–2, R. E. Wheeler, www.r-project.org). All the linear models were tested with a limit of 5000 permutations using an estimate of p-values that stops the sampling when the estimated standard deviation of the p-value falls below a threshold (lmPerm manual, R.E. Wheeler, www.r-project.org); testing for normality and to ensure that the results were not driven by outliers, we used a leave-one-out cross-validation and Cook's distance [36] while gender differences were estimated using a two-sample Student's t-test. All statistical estimates were covaried with the scanner data. Correlations were deemed significant and reported if p≤0.05 (corrected).

## Results

**Figure 2:**
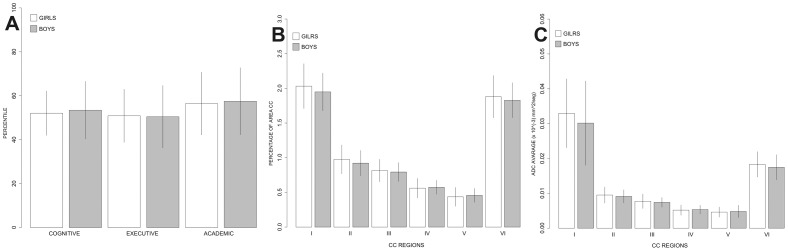
A) Average score obtained for each component of the neuropsychological assessment of children (ENI). B) Corpus callosum area adjusted for mid-sagittal total area brain (TAB). C) Average mid-sagittal ADC in each CC region.

The ENI scores for boys and girls were similar (Figure 2A). The only significant differences found were in writing speed (t_55_ = 2.486, p≤0.016) and constructional abilities with objects (fine motor tasks) (p≤0.034), where girls performed better than boys, and in auditory perception (p≤0.036), where boys performed better than girls. There was a tendency for a larger area in the genu and splenium in girls. Gender differences in each of the CC region areas were not significant, either in the average total area or in the area of each subregion (Figure 2B).

### Correlations between ENI and CC mid-sagittal area

**Figure 3:**
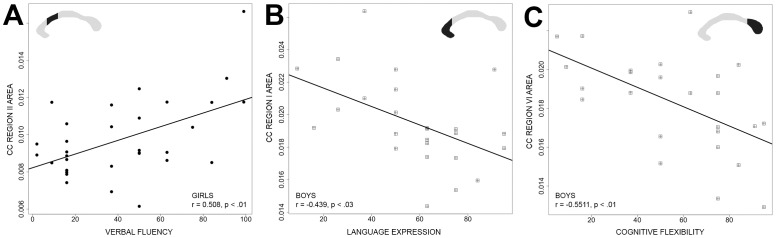
Correlation between ENI and corpus callosum area adjusted for mid-sagittal total area of the brain (TAB). A) Verbal fluency and CC region II. B) Language expression and CC region V. C) Cognitive Flexibility and CC region VI. Girls are represented as black circles, while boys are denoted by squares.

No significant correlations were found between the CC mid-sagittal area over the whole sample and the ENI subtest scores. However, important gender differences were found: CC areas showed a positive correlation with the ENI scores in girls, while the correlation was negative in boys (Table 3). Girls showed a positive correlation between region II area and verbal fluency (r = 0.508, p≤0.002) (Figure 3A). The verbal fluency subtest (executive functions domain) consists in having children say the greatest possible number of words related to fruits, animals, or beginning with a given phoneme, with each category lasting one minute. Boys showed a negative correlation between the area of the genu (region I) and language expression (cognitive functions domain) (r = −0.439, p≤0.0280) (Figure 3B) and between the area of splenium (region VI) and cognitive flexibility (perseverance) (r = −0.551, p≤0.00424) (Figure 3C). In the language expression subtest children must correctly name pictures, utter expressions with a minimal length, and coherent narrative. The cognitive flexibility subtest (executive functions domain) in the ENI is similar to the well-known WCST test (Wisconsin Card Sorting Test) [37]–[38]. The subtest requires that cards with geometric figures be classified with respect to form, number, and color, but the subject is not informed of the classification criterion, and said criterion is changed during the test without telling the subject, which is why the subject must infer it. Within this subtest the child's perseverance is also evaluated; in other words, whether the child persists or not in responding with an incorrect characteristic of the stimulus (shape, color, or number).

**Table 3:** Correlations between neuropsychological assessment of children (ENI) and the CC region area (see figure 1).

CC	GIRLS	BOYS
**I**		Language Expression
		r = −0.4392, p = 0.0280
**II**	Verbal fluency	
	r = 0.5083, p = 0.0029	
**VI**		Cognitive flexibility (perseverance)
		r = −0.5511 p = 0.0042

### Correlations between ENI and CC mid-sagittal ADC values

**Figure 4:**
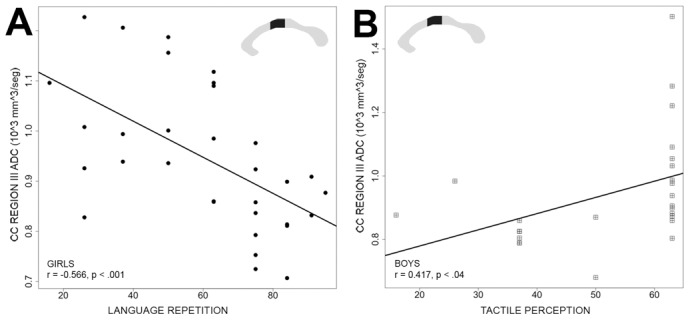
Correlations between ENI and ADC values (mm^3^/s). A) Language repetition and CC region III ADC. B) Tactile perception and CC region III ADC. Girls are represented as black circles, while boys are denoted by squares.

In the age range studied, no significant differences were found between boys and girls in average ADC values measured in each of the CC regions (Figure 2C). Estimates of CC microstructure based on the ADC revealed no significant differences between girls and boys in any of the CC regions, and no significant correlations were found when comparing the whole sample ADC and the ENI subtest scores. However, the ADC values correlated with ENI subtests differentially according to gender (Table 4). Girls showed a negative correlation between ADC in region III of the CC and language repetition subtest (cognitive functions domain) (r = −0.566, p≤0.0007). In this test children must repeat words, pseudowords, and sentences (Figure 4A). Boys presented a positive correlation between ADC in region III and tactile perception (cognitive functions domain) (r = 0.417, p≤0.037) (Figure 4B). In this subtest the child, without looking, must identify and name different objects (e.g. comb, key, ring, etc.) by touching them with his/her right or left hand, depending on the indications provided. Interestingly, in the age range studied, within both the ADC (microstructure) and the area (structure), differential correlations based on gender were observed, despite the similarities in both their cognitive performance and their overall CC characteristics.

**Table 4:** Correlations between neuropsychological assessment of children (ENI) and ADC of the CC region (see figure 1).

CC ADC	GIRLS	BOYS
II		Planning design
		r = −0.4499, p = 0.0240
III	Language repetition	Tactile perception
	r = −0.5666, p = 0.0007	r = 0.4172, p = 0.0379
V		Logical mathematical reasoning
		r = −0.5731, p = 0.0024
VI		Logical mathematical reasoning
		r = −0.5913, p = 0.0081

## Discussion

The main findings of this study are, first, that the total CC area varies among subjects, but it does not differ between boys and girls in the particular age group studied (7–9 years of age). Second, the areas of CC sub-regions correlate differentially with cognitive tasks in boys and girls, suggesting functional differences in interhemispheric communication between genders. In the sample studied, strong correlations were found among the areas of regions I, II, and VI, while adults show a positive correlation between the area of the posterior body of the CC and the isthmus [5]. The ADC also correlated differentially with other ENI subtests according to gender, but in both cases this coincided with finding correlations in the anterior section of the CC (region III). We found variation among subjects in total CC area, which agrees with a report by Giedd et al. [2]. When analyzing the cross section by regions, we found that the areas of the 6 CC regions that we used were similar in boys and girls, except the genu and splenium, which tended to be larger in girls. Although this tendency was not statistically significant, a significant difference between genders has been found in adolescents and adults [19]–[24]. The fact that CC regional areas presented gender-differentiated correlations with cognitive processes may be due to temporal differences in the neurodevelopment of white matter, and to functional differences between genders [39]–[45]. Different CC regions develop with individual time courses [2], [24]. In particular, girls presented a significant correlation of verbal tasks with both the CC region II area and the ADC of region III, tasks that are considered a feminine cognitive strength [39], [42]–[45]. Furthermore, fibers passing through region II interconnect the premotor and supplementary motor cortex, and these cortical regions are related to language production [46]. Among adults, the frontal cortical region is mainly associated with cognitive flexibility tests [47]–[51], but we found an association between a subtest of the cognitive flexibility domain with the posterior CC region in boys, which may be explained by the visual dependency school age children present when solving these geometric tasks [52], since such regions process and integrate visual information. The cognitive flexibility subtests of the ENI require considering geometric figures with respect to form, number, and color, and changing strategies are needed in order to answer the items. It is known that fibers crossing through region VI of the CC interconnect the temporal, parietal, and occipital cortices; nevertheless, the fact that these regions of the CC (I and VI) correlated with a better cognitive performance also indicates that in the age range studied in boys, thinner regions of the CC are more efficient in performing interhemispheric communication.

In terms of the microstructural characteristics of WM, the correlation between the ADC values of region III and the scores on the language repetition subtest in girls and with tactile perception in boys can relate to the role of this region in interconnecting the prefrontal cortex (PFC). Broca's area is associated with language production and with the primary motor cortex, which participate in the fine motor movement required for object recognition and planning of movements necessary for the planning designs subtest [47].

Finding significant correlations only in some regions of the CC may also be due to functional differences between men and women and the fact that, when faced with the same task, men and women of similar cognitive abilities seem to use different cognitive strategies and neuroanatomical resources for its resolution [53]. Neuroimaging CC studies have been performed, mostly with adults and very few with children [39]–[42], [53]. Even fewer significant gender differences have been found among children in cognitive abilities. Girls are better at oral expression, language comprehension, and sensory perception, and boys are better at tasks of visual perception and in spatial abilities [54]. We found significant differences between boys and girls: girls performed better than boys in writing speed and construction abilities, while boys performed better than girls in auditory perception. Most literature on gender differences refers to differences in linguistic, mathematics, and spatial abilities, and some compares fine motor skills between men and women [39]–[44], [53]–[55]. Our data seem to reinforce the idea that boys and girls use different neurological resources when performing cognitive tasks. Adults show a lower average ADC in the genu and splenium in comparison to the CC body [22]; this differs from our results and may be due to the fact that children have not finished the CC fiber myelination process. In healthy tissue low ADC values are associated with increased barriers to transverse diffusion, such as a greater axonal density or myelination were associated with better WM integrity [12]–[13], which leads us to suppose that the greater integration of said callosum regions may favor each gender differently. This supposition does not apply for region III of the male CC, since its relation to the tactile perception (III) subtest was positive.

Our study partly confirms previous results [6]–[9] concerning the negative and positive associations between the area of some CC regions and IQ in children. However, we did not use a global measuring system, such as IQ, but rather an instrument that allows for an assessment of neuropsychological cognitive abilities and therefore allows precise correlations. Our results lead us to think that, although girls and boys have similar CC structural and microstructural characteristics in general, they use their cognitive resources differently to solve the same task. In addition, we suggest that children with a thinner cross sectional area show better cognitive performance, suggesting that in healthy children, thinner cross sectional areas are related to greater efficiency. However, the type of task where certain CC characteristics are associated with cognitive abilities in developing subjects was different for each gender. Other differences between genders observed in fMRI, cognitive abilities, and neurodevelopment in previous studies with adults indicate that the gender-differentiated correlations may be due to differences in interhemispheric communication, resulting in a differential dependency by gender on the CC for cognitive functioning.

Manual segmentation of the CC using a fixed region scheme and not a functional or structural, subject-specific scheme is a limiting factor of this study. For example, sub-segmentation of the CC based on tractography could result in a better CC region classification based on cortical connectivity of each subject [4], [56]. One of the main contributions of this study is the examination of interhemispheric connectivity and its relation to cognitive function by using a neuropsychological test that can evaluate different cognitive domains instead of a test that provides a global score like the IQ. Although this is an improvement, any psychometric measurement has limitations due to the complexity of evaluating cognitive processes at any age. Nonetheless, our findings are relevant for differences between genders in the relationship between the CC and cognitive abilities evaluated with a standardized instrument design for a specific population that explores diverse cognitive abilities in the cognitive, executive, and academic domains.

In summary, although overall CC size and ADC were similar between boys and girls, values of these parameters evaluated for specific CC regions, correlated differently with particular cognitive abilities for each gender. With respect to the CC cross sectional area, we observed a tendency of the cognitive abilities to correlate with the anterior portions of the CC. Our results also point to the need for broader studies on the relationship between specific cognitive processes and structural and microstructural characteristics of the CC in healthy children. The present study contributes to understanding how the CC is related to cognitive abilities during development in each gender by studying a large sample of children that were carefully screened using the ENI, an instrument that gives a richer panorama of cognitive function than standard tests.

## Supporting Information

Table S1 **Description of the Categories, subtests and tasks present in the ENI; NEUROPSYCHOLOGIAL ASSESSMENT OF CHILDREN TEST, (ENI, from the Spanish “EVALUACIÓN NEUROPSICOLÓGICA INFANTIL”).** Matute E, Rosselli M, Ardila A, Ostrosky-Solís F (2007) Evaluación Neuropsicológica Infantil [Child Neuropsychological Evaluation]. Manual Moderno UNAM: Univiversidad de Guadalajara, México.
(DOCX)
