# Supplementary material for: Correlation between Corpus Callosum Sub-Segmental Area and Cognitive Processes in School-Age Children
Source: PLoS One. 2014 Aug 29;9(8):e104549. doi: 10.1371/journal.pone.0104549 (PMC4149349; doi:10.1371/journal.pone.0104549)
Supplement: Table S1 — Description of the Categories, subtests and tasks present in the ENI; NEUROPSYCHOLOGIAL ASSESSMENT OF CHILDREN TEST, (ENI, from the Spanish “EVALUACIÓN NEUROPSICOLÓGICA INFANTIL”). Matute E, Rosselli M, Ardila A, Ostrosky-Solís F (2007) Evaluación Neuropsicológica Infantil [Child Neuropsychological Evaluation]. Manual Moderno UNAM: Univiversidad de Guadalajara, México. (DOCX) [file pone.0104549.s001.docx]

| **CATEGORY** | **SUBTEST** | **TASKS** |
| --- | --- | --- |
| **COGNITIVE FUNCTIONS** | | |
| **Construction abilities** | With objects | Build figures with toothpicks following a graphic model. |
|  | Graphic | Draw a human figure. |
|  |  | Copy composite geometric figures. |
|  |  | Copy a complex composite figure made up of various lines, squares, and triangles |
| **Memory** | Verbal and Visual (Codified) | Remember a list of words heard previously, repeat them afterwards and differentiate them audibly. |
|  |  | Remember figures seen on an image, draw them afterwards and recognize them visually. |
|  | Verbal and visual (differed) | After approximately half an hour the child must remember the figures and words shown (draws and says), spontaneously and grouping them by category |
| **Perception** | Tactile | Recognize objects (e.g. spoon, key, ring) with the right and left hand (alternatively and randomly) without looking. |
|  | Visual | Visually perceive images on slides and identify the superimposed images and on slides with blurry images. Identify objects in incomplete images (visual closure). |
|  |  | Recognizing emotional states in pictures of human faces with expressive gestures. |
|  |  | Object integration. |
|  | Auditory | Listen to pairs of musical notes and identify if they’re the same or different. |
|  |  | Listen to environmental sounds and identify them. |
|  |  | Listen to two syllable words which are phonetically similar and identify if they’re the same or not. |
| **Language** | Repetition | Oral repetition of: syllables, words, non words, and sentences. |
|  | Expression | Expressing the word that corresponds with the image. |
|  |  | Coherently explain a previously read story. |
|  |  | Length of written speech. |
|  | Comprehension | Point to the image that corresponds to the words. |
|  |  | Following instructions. |
|  |  | Understanding speech. |
| **Meta-language** |  | Phonemic system. |
|  |  | Counting sounds and words. |
|  |  | Spelling out words. |
| **Spatial abilities** |  | Correctly identify and express left and right |
|  |  | Right-left expression. |
|  |  | Identify the spatial orientation of drawings from different angles. |
|  |  | Identify the orientation of lines located in a figure made up of lines in a different spatial orientation. |
|  |  | Locate coordinates on a map. |
| **Attention** | Visual | Cancel drawings with slight differences from the rest. |
|  |  | Cancel letters with a specific accompaniment (e.g. A, only if it comes after X). |
|  | Auditory | Repeat digits in direct and inverse order. |
| **Conceptual abilities** |  | Identify similarity in concepts with concrete differences. |
|  |  | Matrixes. Identify the missing fragment of a graphic matrix. |
|  |  | Mentally solve arithmetic problems without using paper and pencil. |

| **CATEGORY** | **SUBTEST** | **TASKS** |
| --- | --- | --- |
| **ACADEMIC PERFORMANCE** | | |
| **Reading** | Precision | Dictation: syllables, words, no-words, and sentences. |
|  |  | Correct and wrong answers when writing down a story the child hears. |
|  | Comprehension | Sentence comprehension. |
|  |  | Comprehension of a text read to the child. |
|  |  | Comprehension of a text the child reads silently. |
|  | Speed | Reading outloud. |
|  |  | Reading silently. |
| **Writing** | Precision | Syllable, word, no-word, and sentence dictation |
|  |  | Correct and wrong answers when writing. |
|  | Narrative composition | Narrative coherence of a text elaborated by the child. Length of his/her narrative production |
|  | Speed | Speed when copying a text. |
| **Arithmetic** | Counting | Counting figures. |
|  | Numerical management | Reading numbers |
|  |  | Dictation of numbers. |
|  |  | Comparison of written numbers (<, >). |
|  |  | Organizing quantities in ascending order. |
|  | Calculus | Mental calculation of numbers in direct or indirect series (3 by 3). |
|  |  | Mental calculation of mathematical operations (addition, subtraction, multiplication, division, fractions). |
|  |  | Written calculation of mathematical operations (addition, subtraction, multiplication, division, fractions). |
|  | Logical-mathematic Reasoning | Solving arithmetic problems. |
| **EXECUTIVE FUNCTIONS** | | |
| **Cognitive fluidity** | Verbal | Verbal fluidity of words in the following categories: Fruits and Animals. |
|  |  | Phonemic (/m/ sound). |
|  | Graphic | Semantic fluidity (drawing things, animals or geometric figures). |
|  |  | Non-semantic fluidity (making different figures with 4 lines that cross through the middle of the frame). |
| **Cognitive Flexibility** | Perseverance | Classifying cards without any prior instructions. The subject must identify the rule of the game through the tester’s oral feedback (he/she only indicates correct or incorrect). Said rule changes without previous warning and the subject must deduce the new rule. Perseverance or not in indicating a non-succesful response. |
|  | Categories | Identify the rule of the game in order to classify the cards in the following categories: color, shape and number. |
|  | Organization | Not using an unsuccessful response after five correct responses. |
| **Planning & organizing** | Designs | Number of designs that were correctly built according to the rules and the pyramid model. |
|  | Movements | Correct and minimal movements performed to build the solicited pyramid model. |
